# Two-photon fluorescence lifetime for label-free microfluidic droplet sorting

**DOI:** 10.1007/s00216-021-03745-2

**Published:** 2021-11-18

**Authors:** Sadat Hasan, Maximilian E. Blaha, Sebastian K. Piendl, Anish Das, David Geissler, Detlev Belder

**Affiliations:** Institute for Analytical Chemistry, Leipzig University, Linnéstraße 3, 04103 Leipzig, Germany

**Keywords:** Microfluidics/microfabrication, Fluorescence lifetime, Two-photon excitation, Droplet sorting, Cell sorting, Label-free fluorescence detection

## Abstract

Microfluidic droplet sorting systems facilitate automated selective micromanipulation of compartmentalized micro- and nano-entities in a fluidic stream. Current state-of-the-art droplet sorting systems mainly rely on fluorescence detection in the visible range with the drawback that pre-labeling steps are required. This limits the application range significantly, and there is a high demand for alternative, label-free methods. Therefore, we introduce time-resolved two-photon excitation (TPE) fluorescence detection with excitation at 532 nm as a detection technique in droplet microfluidics. This enables label-free in-droplet detection of small aromatic compounds that only absorb in a deep-UV spectral region. Applying time-correlated single-photon counting, compounds with similar emission spectra can be distinguished due to their fluorescence lifetimes. This information is then used to trigger downstream dielectrophoretic droplet sorting. In this proof-of-concept study, we developed a polydimethylsiloxane-fused silica (FS) hybrid chip that simultaneously provides a very high optical transparency in the deep-UV range and suitable surface properties for droplet microfluidics. The herein developed system incorporating a 532-nm picosecond laser, time-correlated single-photon counting (TCSPC), and a chip-integrated dielectrophoretic pulsed actuator was exemplarily applied to sort droplets containing serotonin or propranolol. Furthermore, yeast cells were screened using the presented platform to show its applicability to study cells based on their protein autofluorescence via TPE fluorescence lifetime at 532 nm.

**Graphical abstract:**

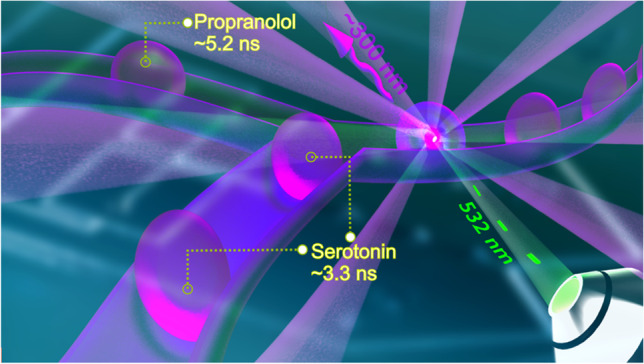

**Supplementary Information:**

The online version contains supplementary material available at 10.1007/s00216-021-03745-2.

## Introduction

Droplet microfluidics involves nano-to-femtoliter compartmentalization by emulsions [1] to segregate desired fluids or entities dispersed or dissolved in the fluids [2]. Droplet microfluidics is a powerful tool in various application areas such as single-cell analysis [3], small–scale cell cultures [4], chemical synthesis [5, 6], high-throughput drug screening [7, 8], and nanodevice fabrication [9]. The ability to sort droplets of choice for further processing, commonly referred to as *droplet sorting*, is a prime tool of the microfluidic toolbox [10–14].

In microfluidic droplet sorting, a crucial aspect is the detection technique to trigger the actuation process. In its early stage of development, detection via fluorescence intensity was established and referred to as fluorescence-activated droplet sorting (FADS) [15].

FADS is probably difficult to beat in terms of sensitivity and readout speed, but the analytes have to be tagged by fluorescent dyes in a prior process [9, 14, 16]. While fluorescent labeling is well-established and works well for many biomolecules, it complicates the workflow [17]. Furthermore, it limits the application range, as there are many applications where fluorescent labeling is difficult [18] or even impossible.

That is why label-free detection alternatives have been long sought, and significant efforts have been reported, such as UV–Vis spectra [19], optical absorbance [20], Raman scattering [21], magnetic detections [22], mass spectrometry [23], and image recognition [10, 24, 25]. Some of these detection techniques, such as Raman or mass spectrometry, also allow samples to be analyzed with high chemical specificity. In this context, mass spectrometry [23] excels, but the technique is destructive and also relatively slow. Accordingly, the search is still on for non-destructive, label-free detection techniques that have a high chemical specificity and, at the same time, are fast enough to allow real-time actuation.

Even though classical fluorescence detection does not meet these requirements, there are ways to empower fluorescence detection in terms of being label-free and having higher information content. This can be achieved by shifting the excitation wavelengths from the typical visible range to the UV range and by recording fluorescence lifetime rather than just intensity. If the excitation wavelength is shifted from the typical visible range to the deep-UV range, the application is no longer limited to selected fluorescent dyes. For example, structures containing an aromatic ring often show an absorption maximum in the deep-UV region (~ 260 nm) [26], which enables autofluorescence-based detection of various species from small aromatics [26, 27] to cells [28, 29].

However, on-chip implementation of autofluorescence using deep-UV is challenging. The chip material and the optical setup must be transparent for deep-UV, hence limiting the chip substrate to fused silica or quartz glass. Additionally, regarding living cells, high phototoxicity is problematic [30]. There is an elegant way to resolve these shortcomings of deep-UV excitation by applying two-photon excitation (TPE). For example, a typical 266-nm deep-UV source translates to excitation at 532 nm in the visible range. This is perfectly compatible with common chip materials [31–34] and optical elements, which also leads to less photostress in living cells [35–37].

As stated above, the information content of intensity-based fluorescence is limited and unable to distinguish different compounds with similar fluorophores. This can be achieved by determining the fluorescence lifetimes, e.g., by the time-correlated single-photon counting (TCSPC) technique [12, 38]. It is also compatible with droplet sorting as shown in earlier work [39]. Fluorescence lifetime–activated droplet sorting (FLADS) has so far only been applied using traditional single-photon excitation in the visible range (470 nm), and is therefore restricted by the same limitations in terms of label-chemistry as discussed above.

This is the starting point for the present work, in which we investigate whether FLADS can be extended by using two-photon excitation. With such a two-photon fluorescence lifetime–activated droplet sorting (TPE-FLADS) technology, it should be possible to sort droplets according to their different chemical and biochemical content in a label-free and information-rich manner. In principle, it should even be possible to sort cells without labeling, which would allow us to explore a broad range of new applications.

To this end, we develop a droplet generation and sorting platform that allows for the sorting of aromatic compounds such as propranolol and serotonin based on their fluorescence lifetimes and, as a proof of concept, screening of live and dead yeast cells, without labeling.

## Materials and methods

### Microfluidic chip design and fabrication

**Fig. 1:**
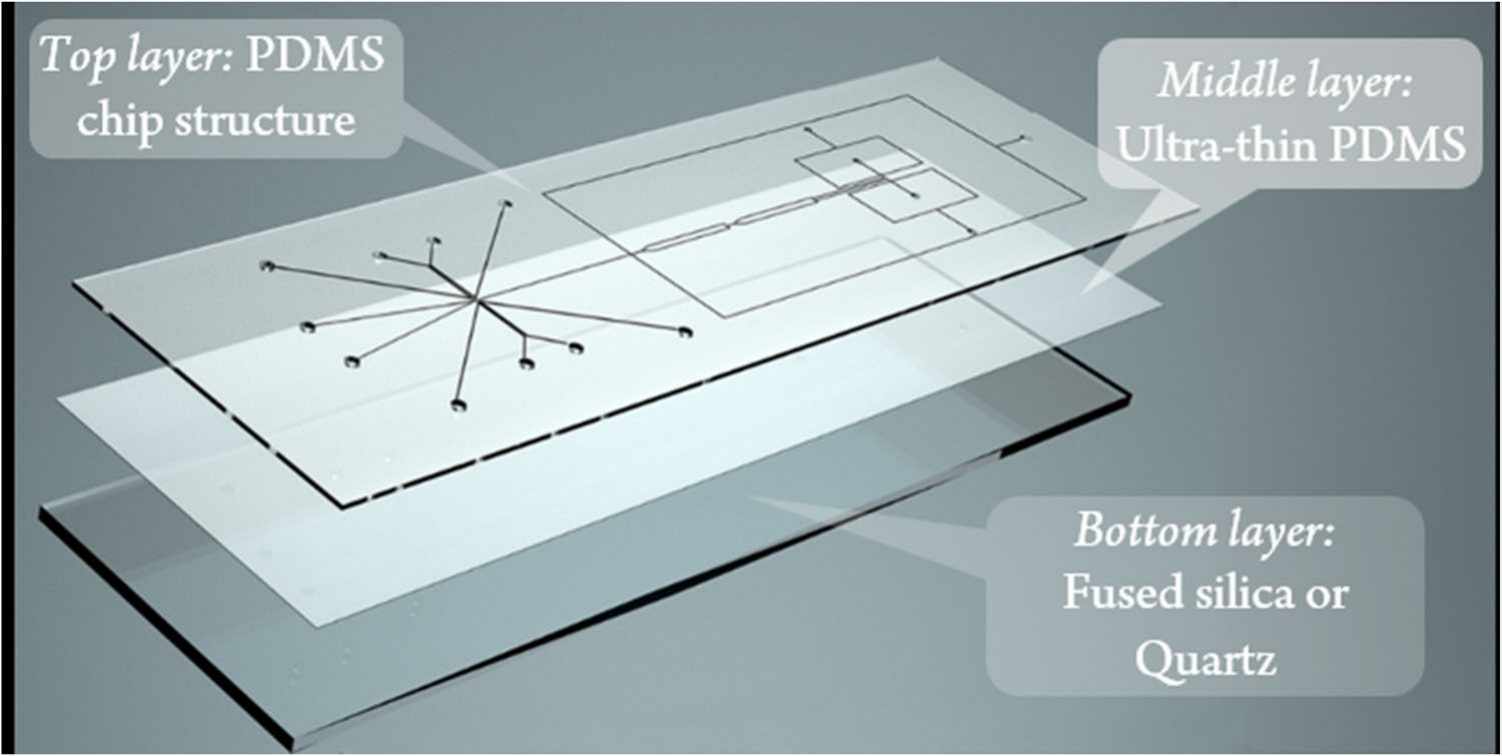
Schematic drawing of the newly developed three-layer chip system consisting of a microstructured PDMS slide bonded on top of a PDMS-coated fused silica slide

The microfluidic chips used throughout this work were designed and manufactured in-house and consisted of three layers. While the microfluidic channels were integrated into the upper polydimethylsiloxane (PDMS) layer (Dow Corning, USA), the bottom side consists of an ultrathin PDMS layer deposited on an FS/quartz glass slide. Figure 1 provides a visual description of the newly developed chip design, including the layers.

**Fig. 2:**
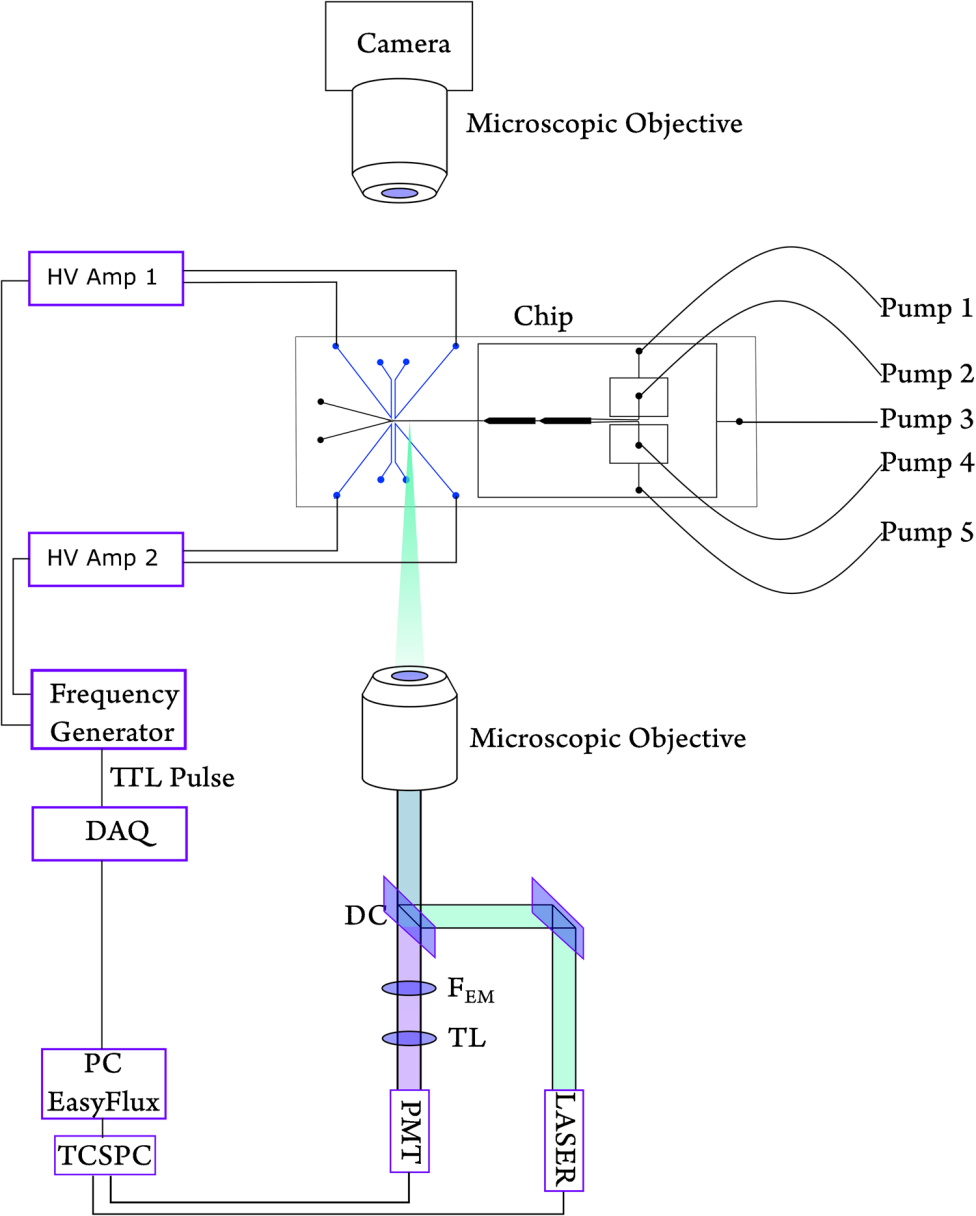
Experimental setup, including the microfluidic chip, optics, and the fluidic system. DC dichroic mirror, FEM emission filter, TL tube lens. Pumps 1–5 are connected to individual modules of the pressure-driven pump system

The chip layout (Fig. 2) consisting of two flow-focusing droplet generators (100 μm width, 75 μm depth), two storage and droplet stabilization chambers (1 mm width, 75 μm depth), an oil spacing channel (100 μm width, 75 μm depth), and two droplet sorting channels (110 μm width, 75 μm depth) was designed as a vector graphic using the open-source software Inkscape (version 0.92, www.inkscape.org). After printing an inverted image (i.e., channel parts are transparent and other parts are blackened) on a transparent foil (10,000 dpi, DTP-system Studio, Germany), manufacturing of the top PDMS layers could be realized according to our previous work [39]. Briefly, the top microstructured PDMS layers were molded on silicon wafers fabricated by conventional photolithography.

For the bottom layer, an ultrathin PDMS layer (< 20 μm) was spin-coated at 4500 rpm for 60 s on top of FS (76 × 26 × 0.25 mm and 76 × 26 × 1 mm, Siegert Wafer, Germany) or quartz glass (75 × 25 × 0.5 mm Plano, Germany). For substrates of 500 μm thickness, quartz was used instead of FS due to commercial availability and since FS and quartz provide almost similar optical transmission in the UV band. The top layer (PDMS) with channel structures and the bottom layer are bonded together using oxygen plasma at 1.5 mbar pressure after 30-s activation. After bonding, the electrode channels are filled with an alloy with a melting point at 58 °C (Sonderweichlot 302, S-Bi49ln21Pb18Sn12, Chemet, Germany) with the help of a conventional hot plate at 75 °C.

### Chemical preparation and fluidic instrumentation

Propranolol and serotonin solutions were prepared from propranolol hydrochloride and serotonin hydrochloride (both from Sigma-Aldrich, Germany) with ultrapure water (TKA Wasseraufbereitungssysteme, Germany) in different concentrations freshly before the experiments. The oil phase is Novec HFE 7500 (3 M, Germany) with 2% surfactant of Pico-Surf 1 (Sphere Fluidics, UK) for droplet stabilization.

All fluids were pumped into the chip using high-precision pressure-driven pumps (Fluigent, Flow EZ, France) via PTFE tubing (1.58/0.3 mm outer/inner diameter, Sigma-Aldrich, MO, USA). The aqueous phases (two individual modules) were pumped by pressure modules capable of pumping up to 345 mbar. For the oil phases, pressure modules (three individual modules) of a maximum of 1000 mbar were used. During the experiment, pressure in the aqueous and the oil phase was 50 ± 10 and 300 ± 50 mbar ranges, respectively. The pressure was controlled locally at the pressure module.

### Yeast cell preparation

Five milligrams of commercial budding yeast was diluted in 1 ml of isotonic PBS (pH 7.4) (Merck, Germany) containing 1 g/l glucose (Merck, Germany). The cells were centrifuged at 2900* g* for 1 min and resuspended in fresh PBS buffer three times for washing using a standard benchtop centrifuge (VWR Galaxy 14D, USA). The suspension was split into two batches, where one batch was heated overnight at 70 °C using a thermomixer (Eppendorf, Germany) at 500 rpm, referred to as the dead yeast cells. The other batch was preserved at room temperature, referred to as the live cells. For detection, the cell suspension was diluted step-by-step to obtain a concentration of approximately 10 cells per droplet.

### Optical setup

The laser module consists of an Nd:YVO_4_ laser source (Cougar, Time-Bandwidth Products, Switzerland). It produces 1064 nm at the source, and by frequency-doubling, we achieve the excitation wavelength of 532 nm. The laser is pulsed at a 20-MHz repetition rate, and the pulse width is 12 ps. The PicoHarp 300 (PicoQuant, Germany) device was used for TCSPC measurements. The excitation beam enters the microscope (Olympus IX71 inverted microscope, Germany) through a dichroic mirror (ZT532SPRDC, short pass 532 nm, AHF Analysentechnik, Germany) and illuminates the chip through the objective (60 × , UPLANSApo water immersion, numerical aperture (NA) = 1.2, WD = 280 μm, Olympus or 40 × LUCPlanFLN, NA = 0.6, WD = 3.00–4.20 mm, Olympus, Germany). The fluorescent emission passes the same lens and is filtered by a bandpass filter of 295–385 nm (DUG 11, Schott, Germany) right after the dichroic mirror. A photomultiplier tube (PMA 165 nm, PicoQuant, Germany) detects the emission. The droplet sorting event was captured by a customized upright microscope, as illustrated elsewhere [39].

### Electronic setup

The TCSPC output is carried out to a previously developed LabVIEW (National Instrument, USA)-based program “EasyFlux.” It is available as an open-source program (https://github.com/SadatHasan/EasyFlux). According to the average lifetime threshold, if a droplet fulfills the sorting criteria, the data acquisition (DAQ) device (USB 6000, National Instrument, USA) generates a transistor-transistor logic (TTL) pulse. The DAQ pulse triggers the signal generator (Rigol Technologies, DG 1032 dual-channel, China) to provide 10–15 V in 1–1.5 kHz sine-wave as output.

We configured the signal generator to function in triggered mode. The same DAQ output goes to the first and the second trigger channels. The first channel of the signal generator is activated by a TTL high pulse from the DAQ. A TTL-low activates the second channel. The channels of the signal generator at the end drive the embedded electrodes on the chip. The first channel drives the sorting-side electrodes, and the second one is for the waste side. To ensure that all unwanted droplets go to waste, waste channel electrodes are designed to be always activated. Whenever there is a TTL high signal due to the desired droplet’s detection, the sorting electrodes are activated, and simultaneously, the waste electrode is deactivated. Two independent high-voltage amplifiers (Trek-2210, Trek, USA) amplify the signal generator outputs with a fixed gain of 100. One amplifier output triggers the sorting channel electrodes, and the other one triggers the waste channel side electrode pair. The electronic signals (from DAQ output until amplifier output) are observed in real-time using a digital memory oscilloscope (GDS-3354, GW-Instek, Taiwan).

We provided a graphical description of the instrumentation in the Fig. 2.

## Results and discussion

To implement droplet sorting, activated by time-resolved TPE fluorescence lifetime, a central challenge is to develop a suitable chip system. In principle, such a microfluidic chip has to be suitable for droplet microfluidics and should enable the sensitive detection of fluorescence in the tiny microfluidic channels. A typical chip material used for droplet microfluidics is polydimethylsiloxane (PDMS). It possesses uniformly hydrophobic channel surfaces, which allows for ideal wetting of the chip material with the continuous oil phase. Furthermore, the manufacturing of PDMS chips is straightforward applying established soft lithographic techniques. For the intended label-free fluorescence detection, however, a crucial requirement is high optical transparency for both the excitation and the emission wavelength. This is not only true for excitation at 266 nm in a one-photon approach but also for the detection of light at higher wavelengths for the two-photon equivalent at 532 nm. Previous studies on label-free detection utilizing PDMS chip materials revealed that full-body PDMS devices are not suitable for this purpose [32].

**Fig. 3:**
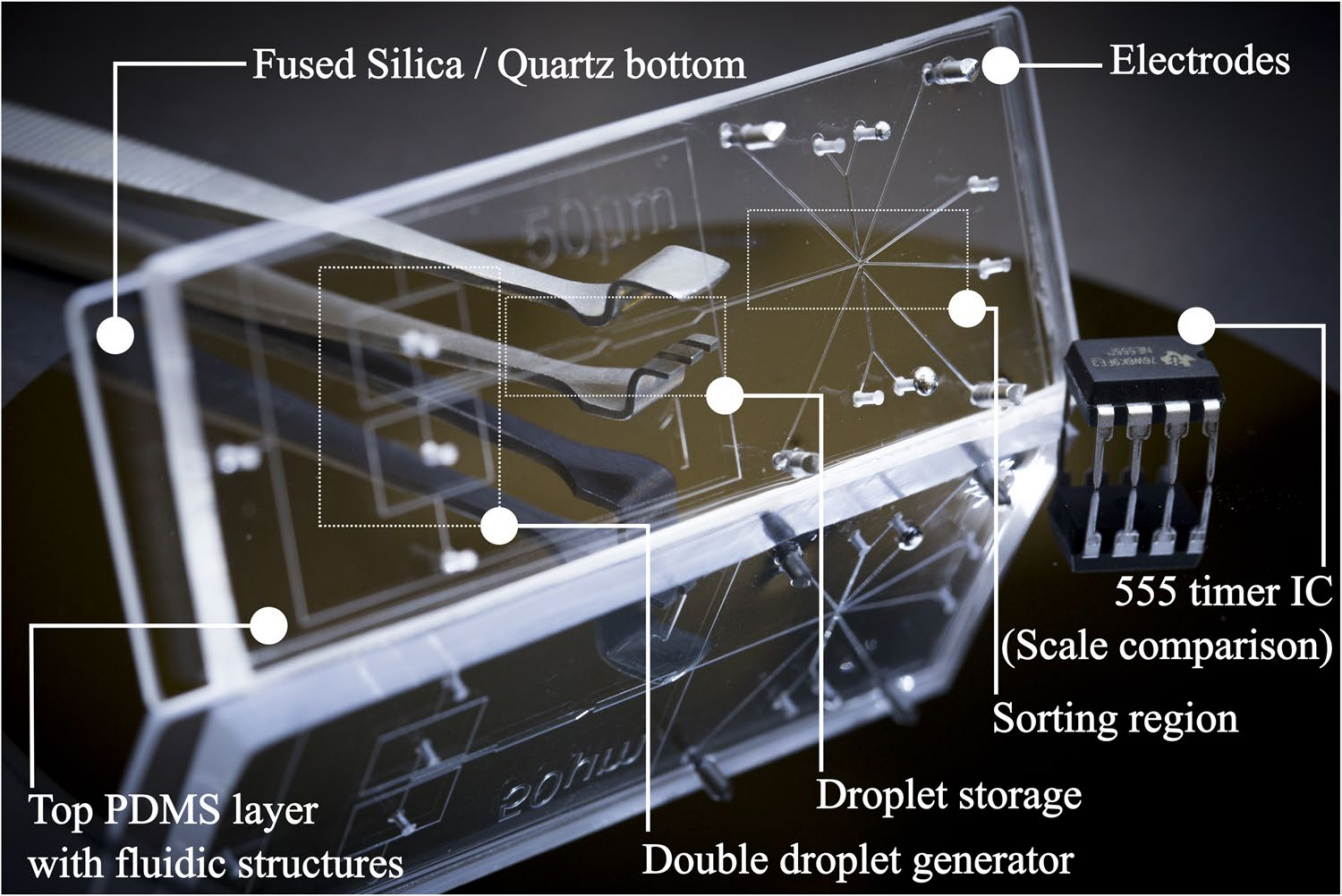
An exemplary view of the chip, developed for TPE fluorescence detection combined with droplet sorting. The chip has embedded electrodes for DEP droplet actuation. An electronic integrated circuit (IC) of ~ 1 cm length is presented for scale comparison

Therefore, we have developed a 3-layer hybrid chip with high UV transparency [40] and homogeneous hydrophobic properties. The layout consists of a PDMS-top layer with the microfluidic structure and a PDMS sublayer (< 20 μm) spin-coated on a fused silica slide. A schematic of the chip layout and the layering is shown in Fig. 1. This allows several things to be achieved at once: having uniformly hydrophobic PDMS channels for reliable droplet microfluidics and the highest possible transparency in the UV range, while ensuring high mechanical stability of the chip. In Fig. 3, an exemplary image of the built chips is presented.

We started with a set of experiments using serotonin droplets of 1 mM generated at 1 Hz to assess the general suitability of the system. In preliminary experiments, it became clear that the photon count must be maximized for valid TCSPS measurements in individual droplets [41]. In addition to the transparency of the chip, other elements in the optical assembly and ray path also contribute to this, which we investigated in the course of the study. The amount of fluorescent light collected is also determined by the choice of objective, and in the case of an inverse fluorescence setup, the thickness of the chip bottom also plays a role. The results on testing various objectives and chip layouts and experimental parameters can be found in the supporting information.

The highest photon counts were achieved with a 60 × immersion objective and the thinnest available fused silica substrates (250 μm), but these combinations proved unfavorable in terms of chip fabrication and handling. The combination of 1 mm FS + 20 μm PDMS chip bottom and a 40 × objective turned out to be the most practicable combination and was used in further experiment chips. Additional investigations were carried out to determine the influence of the droplet frequency and the laser power on the optical gain. A ~ 4.43 mW laser power yields a photon count of ~ 10 Cts/ms. This is sufficient to calculate the average fluorescence lifetime with high accuracy and a standard deviation below 0.05 ns in calculated average fluorescence lifetimes (SI Figure S1A).

**Fig. 4:**
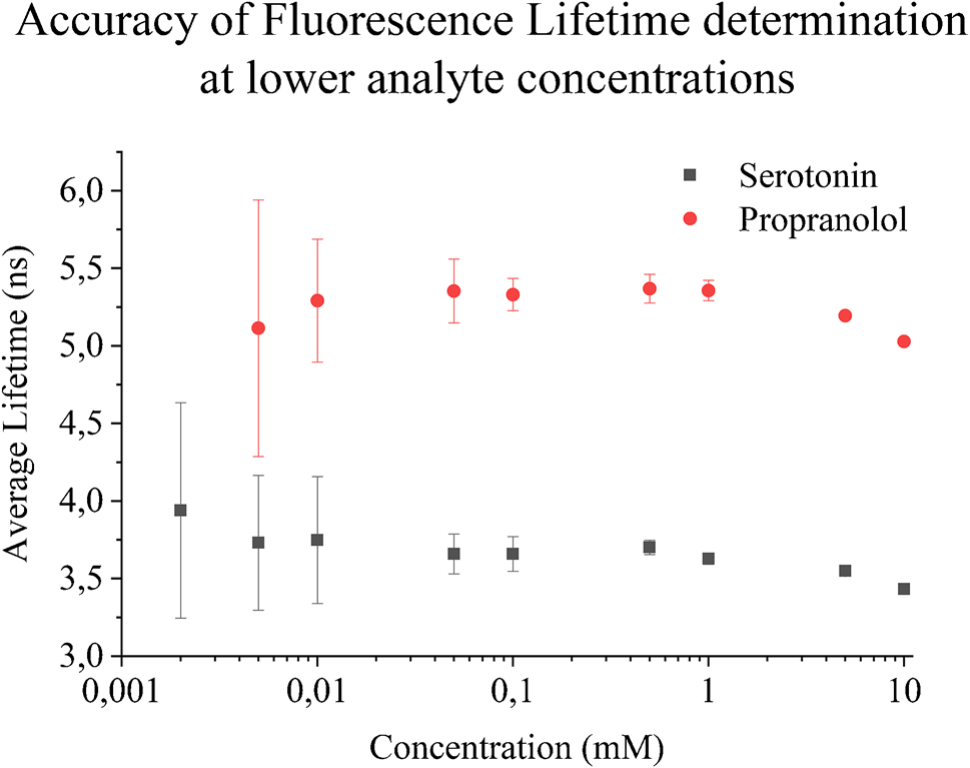
Accuracy of average fluorescence lifetime of serotonin and propranolol in droplets, in other words LoD of serotonin and propranolol concentrations. The experiment was performed using a 250-μm FS + 20 μm PDMS chip using ~ 1 Hz droplet frequency at 13.3-mW laser power via the 60 × lens

In order to determine the limit of detection (LoD) and the accuracy of lifetime calculation, we measured the standard error in the average lifetime of serotonin and propranolol droplets by gradually lowering the concentrations (Fig. 4). Figure 4 refers to the fact that below 50 μM concentration, the standard error is significantly high; therefore, it can be assumed as the LoD for the given conditions.

One last critical parameter to be explored is the throughput of the system, i.e., the number of droplets that can be sorted per second. In droplet microfluidics, the aim is often to attain the highest possible droplet frequency, which is also easier to achieve reliably. In preliminary tests, droplet frequencies of significantly more than 100 droplets per second could be reliably generated with the chip described here. However, there are limits to the droplet frequency when using relatively slow detection techniques for droplet detection such as time-correlated single-photon counting. At the current state, the LabVIEW-based EasyFlux platform needs approximately 10–30 ms to calculate the average fluorescence lifetime of individual droplets [39]. This condition limits the throughput of the system to 20 Hz for highly accurate sorting. However, the influence of the droplet frequency from 1 to 20 Hz has been investigated (SI Figure S2B). Above 10 Hz, the photon count is determined as insufficient. The details are provided in the Supplementary Information.

With these preliminary experiments, the basis has been established for investigating the capability of the now well-characterized system for droplet sorting.

### Droplet sorting

After developing a suitable setup for TPE fluorescence measurements at 532 nm, we explored its application to droplet sorting. Here we chose serotonin and propranolol as model compounds as they are not detectable by typical fluorescence detection using visible light. These compounds exhibit fluorescence only with deep-UV/UV or their multiphoton counterpart excitation. Since both compounds can be distinguished by their fluorescence lifetime, this parameter can be used for the “on-the-fly” recognition of droplets loaded with one of the compounds. This information can then be used to trigger the sorting function.

**Fig. 5:**
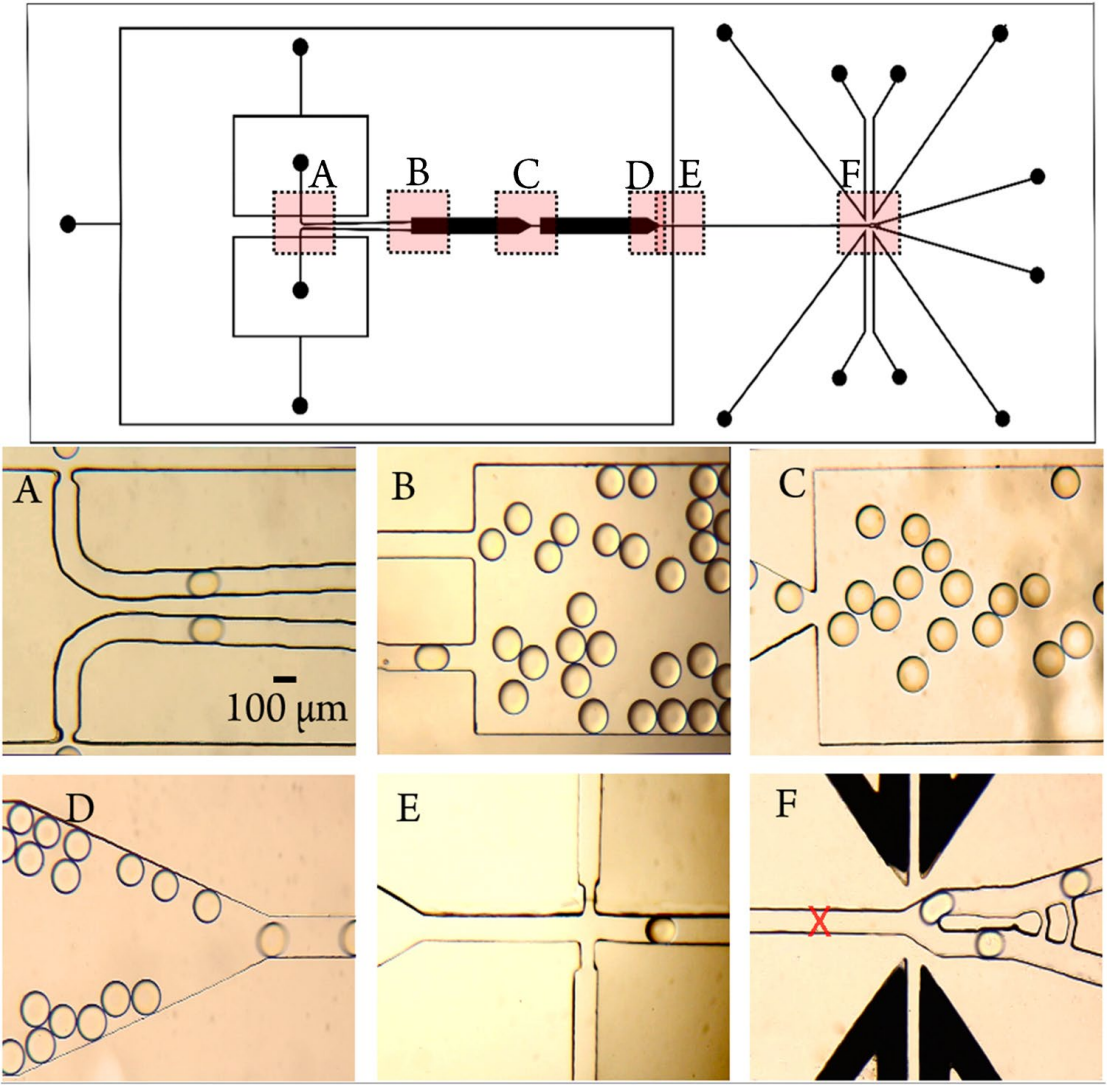
The top segment shows the complete layout of the chip. **A** Double droplet generator, both flow focusing with a 40-μm narrowing structure. **B** Droplets entering first storage structure. **C** Mixing of stabilized droplets. **D** Droplets coming out of second storage. **E** Side oil channels spacing and shooting droplets towards sorting junction in one-after-one fashion. **F** Sorting junction; black channels are the electrodes. Illustrative videos of Fig. 5: **A**–**E** are provided in SI

To test such a droplet sorting system, we need a continuous flow of droplets containing either serotonin or propranolol. For this purpose, we have developed a chip layout that generates droplets containing serotonin and propranolol simultaneously on the device, stabilizes them, mixes them, and then feeds them to a channel where detection and sorting take place. A schematic drawing of the device together with microphotographs of the various functional elements in action is shown in Fig. 5.

Fig. 5A to F show individual images from video microscopy studies that illustrate the function of the multifunctional chip (corresponding video 5A–E in the SI). Figure 5A shows the double droplet generators for simultaneous droplet generation. To avoid droplet coalescence in the merging channel as observed earlier [39], a multi-stage storage chamber was added to the design (Fig. 5B). In combination with the addition of the surfactant (2% Pico-Surf 1in Novec 7500 oil) to the oil phase, the droplet stability is significantly increased due to more prolonged incubation. Consequently, the first storage structure was added to provide sufficient time to stabilize the droplets with surfactants before they come into contact. The second storage structure is to generate random ordering of two different types of droplets and ease of spreading them over large confinement (Fig. 5C and D). Figure 5D shows the droplets exiting the second storage structure, which preferably takes place at a slow flow rate (see Video 5E in the SI). Then, in Fig. 5E, droplets enter the oil spacing crossing one by one. This segment is designed to facilitate adequate droplet spacing and prevent droplet stacking. Finally, after detection by TPE at 532 nm, downstream comes the sorting junction with integrated electrodes, as shown in Fig. 5F. There the droplets are sorted into either the sorting (lower channel) or the waste (upper channel) channel. The dark structures in Fig. 5F are the actuation electrodes generated by filling microfluidic channels with a conductive alloy

Just before the droplet enters the sorting junction (Fig. 5F), it passes over the laser spot (marked by a red cross in Fig. 5F). It leaves its signature fluorescence emission signal, which the PMT picks up. The TCSPC system converts it to electronic data for every single photon and its corresponding arrival time. A histogram visualizing the average photon arrival time for the entire droplet is generated, which allows for the calculation of average fluorescence lifetimes for individual droplets. It takes approximately 10–30 ms to process the lifetime value and generate a trigger pulse to actuate the droplet on-chip.

**Fig. 6:**
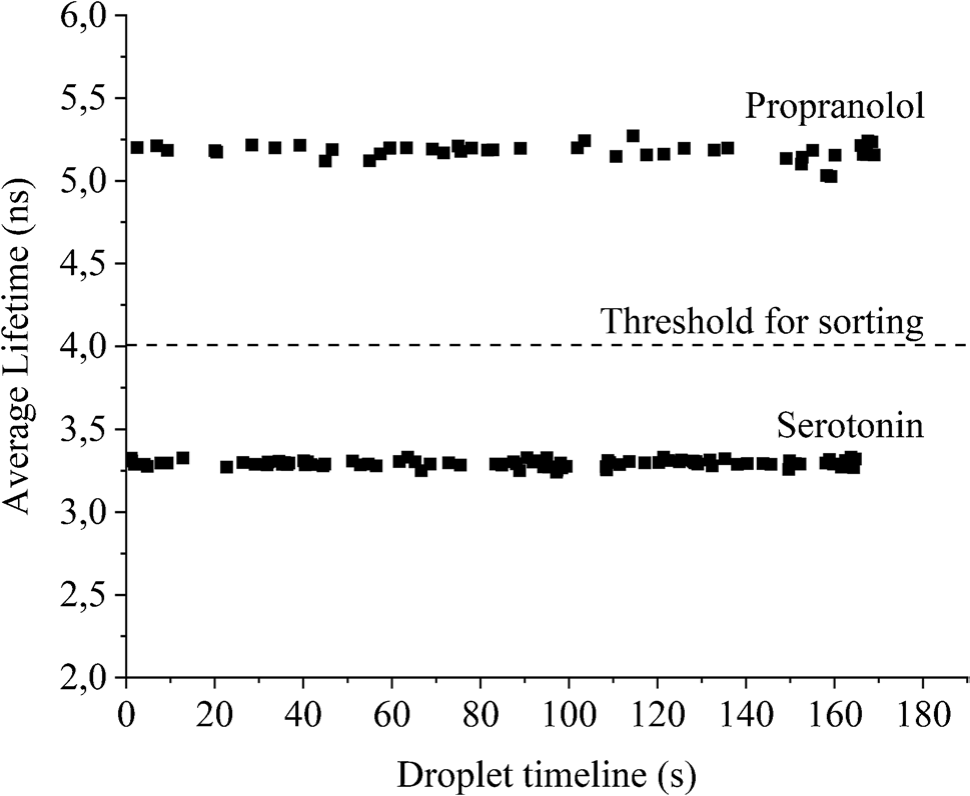
On-chip experiments with serotonin and propranolol resulted in an average fluorescence lifetime of 3.3 and 5.2 ns, respectively. Thus, the threshold is set at 4 ns for sorting

The average fluorescence lifetime of serotonin and propranolol 1 mM solutions was measured in bulk separately and 3.7 and 5.12 ns were recorded, respectively. During the sorting experiment, serotonin and propranolol droplets were observed approximately at 3.6 and 5.2 ns of lifetimes, respectively (Fig. 6). To sort out the propranolol droplets with an average lifetime of approximately 5.2 ns, the threshold is set at 4 ns. Whenever an above threshold droplet is detected, the high-voltage signal activates the electrode pair in the sorting side momentarily, resulting in droplet actuation by the DEP force. Following the DEP pulse, the intended droplet is guided towards the sorting channel. If the lifetime of a droplet is lower than the threshold, which are the serotonin droplets in this case, it goes to the waste channel. For this experiment, the electrode pair in the waste side is always active unless a desired droplet is detected (here, propranolol droplets are the desired droplets as they have above 4-ns lifetime). A video file is provided in the SI (TPE-FLADS Illustrated) to demonstrate the mechanism, where droplets are temporally separated to show real-time playback. The distribution of the average fluorescence lifetime of propranolol and serotonin droplets during a sorting experiment is provided in Fig. 6.

At 1 Hz, the observed accuracy of the sorting system was 100% according to real-time video analysis (as shown in SI video clip TPE-FLADS Illustrated). When approaching 10 Hz, the accuracy of the sorting system was approximately 92%, similar to our previously published FLADS system [39].

This experiment is performed on a chip with a bottom layer of 20 μm PDMS spin-coated on a 1-mm FS slide, combined with a 40 × objective and 4.43-mW laser power at 532 nm, pulsed at 20 MHz repetition rate. Figure 6 shows that with the system developed here, consistent fluorescence lifetimes for on-the-fly detection of droplets using TPE fluorescence detection can be obtained. Thus, the developed system allows for the sorting of droplets containing serotonin and propranolol based on their fluorescence lifetimes in two-photon excitation mode.

### Yeast cell screening

Following these studies with model compounds, we investigated whether this approach could be applied to differentiate and sort single cells in droplets. This would be an exciting new toolbox addition for single-cell analysis, where label-free methods for differentiating cell states are urgently sought. In this proof-of-concept study, we investigated whether on-the-fly differentiation of dead from live cells based on their fluorescence lifetime in two-photon excitation mode at 532 nm is possible. For these experiments, we used a rather straightforward chip design, which just contained one droplet generator. To this end, we first investigated whether the fluorescence lifetimes of dead and living cells in the chip system differ, using yeast cells as model organisms. Initially, the alive and the dead cells were screened separately in droplets wherein an average of 10 cells was encapsulated per droplet. Afterward, we encapsulated a mixture of dead live cells in droplets and screened the mixed cell suspension.

**Fig. 7:**
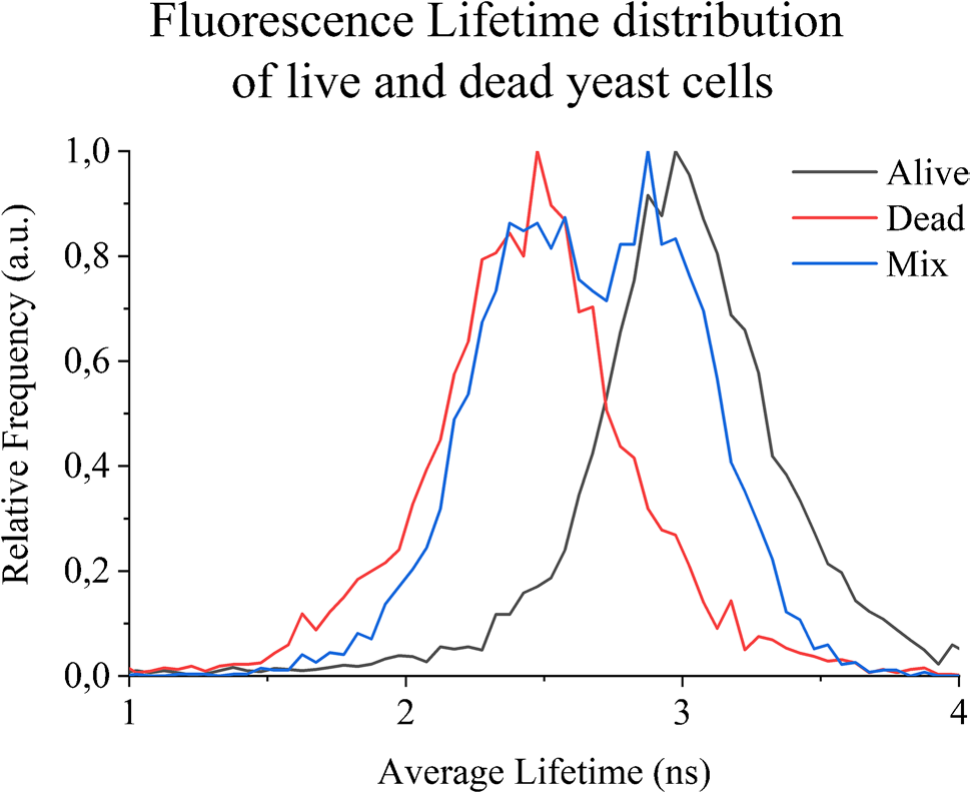
Average fluorescence lifetime distribution of live and dead yeast cells screened via TPE fluorescence lifetime at 532 nm. The dead cells exhibit shorter lifetimes compared to the live ones

To achieve as high photon counts as possible, these experiments were performed with a 250-μm FS + 20 μm PDMS chip bottom in combination with a 60 × objective and 13.3-mW laser power. The results of these preliminary investigations are shown in Fig. 7, which clearly shows that dead and living cells in droplets passing by can indeed be distinguished according to their fluorescence lifetimes.

Due to the denaturation of the proteins caused by the applied heat, dead cells show significantly shorter lifetimes than living cells [42]. Also, in the screened cell mixture, the difference in lifetime is observed with similarity to the individual screening of dead and live cells. However, it is also clear from Fig. 7 that this criterion can hardly be used for unambiguous sorting due to the large variance of the data. This large variation of the fluorescence lifetime data can be explained by the insufficient focusing of the cells in the fluidic channel. Since the excitation focus under two-photon excitation conditions is extremely small, usually less than 1-μm cross-section [43], it leads to very low photon counts if the cells are not being accurately targeted and thus to inaccurate determination of fluorescence lifetimes (SI Figure S3). In order to make the technology described here applicable for single-cell analysis, smaller droplets must be guided accurately and reproducibly through the laser focus. In future studies, this will be investigated in more detail by lowering the channel dimensions and improving the channel geometry to increase the probability of cells’ pathway to meet the laser spot.

## Conclusion

Here, we have demonstrated a novel droplet microfluidic system enabling label-free fluorescence detection of small aromatic compounds and potentially cell screening employing two-photon excitation at 532 nm. Using time-correlated single-photon counting, on-the-fly determination of the fluorescence lifetime of by-passing droplets is made possible. With the help of the fluorescence lifetime signatures, droplets with different chemical contents can now be distinguished. For this purpose, a new three-layer PDMS fused silica chip with an extra thin but hydrophobic bottom was developed to obtain the highest possible photon counts. This allowed us to realize a label-free microfluidic droplet sorting system capable of distinguishing aromatic compounds and the viability of cells by their fluorescence lifetimes. The comparatively low droplet frequencies of 10 HZ in this proof-of-concept study are due to the slow data processing with a desktop PC. By using an embedded system to determine the fluorescence lifetimes, significantly higher frequencies should be possible. The use of quartz glass chips with very thin walls, as can be produced with the selective laser-induced etching (SLE) technique, could also promote the broad application of the technology.

## Supplementary Information

Below is the link to the electronic supplementary material.

Supplementary file1 (DOCX 484 KB)

Supplementary file2 (MP4 1063 KB)

Supplementary file3 (MP4 590 KB)

Supplementary file4 (MP4 776 KB)

Supplementary file5 (MP4 532 KB)

Supplementary file6 (MP4 625 KB)

Supplementary file7 (MP4 2164 KB)
