# Supplementary material for: Two-photon fluorescence lifetime for label-free microfluidic droplet sorting
Source: Anal Bioanal Chem. 2021 Nov 18;414(1):721–30. doi: 10.1007/s00216-021-03745-2 (PMC8748334; doi:10.1007/s00216-021-03745-2)
Supplement: Supplementary file 1 — Supplementary file1 (DOCX 484 KB) [file 216_2021_3745_MOESM1_ESM.docx]

**Two-photon fluorescence lifetime for label-free microfluidic droplet sorting**

Sadat Hasan, Maximilian E. Blaha, Sebastian K. Piendl, Anish Das, David Geissler, and Detlev Belder*

Institute for Analytical Chemistry, Leipzig University, Linnéstraße 3, 04103 Leipzig, Germany.

Supplementary Information

# Video contents

- Video files of different chip segments are provided in the ‘Supporting Information-Video files’ folder, named ‘Video_5A-E.’
- Real-time droplet sorting video, including the EasyFlux front panel, is provided in the same folder. All the experiment settings are visible on the software front panel in the video file ‘TPE FLADS Illustrated.’

# Spincoating protocol

Device: Spin150 Manufacturer: APT GmbH, Bienenbüttel, Germany.

1. 10 mL PDMS with 10% (w/w) curing agent was mixed thoroughly. The air bubbles were eliminated entirely using a vacuum pump. This transparent PDMS was poured from one side to the other (the longer side of the glass slide) on the glass slide (approximately 3-5 mL), ensuring no air bubbles in the liquid PDMS.
2. This glass slide is then placed carefully on the holder of the spincoater.
3. Spincoater settings:

- 4500 rpm
- Acceleration 500 rpm/sec
- Spin time 60 sec
- Vacuum ON

1. After spincoating, the glass slide is placed on a hot plate for 2 hours at 95˚C.

# Intensity of serotonin 1 mM droplets in different conditions

To test the Influence of different microscopic objectives, we have considered a 250 µm FS slide coated with 20 µm PDMS subsequently plasma-bonded with the PDMS top layer containing the channel structures and tested the optical gain of a 40x and 60x objective. As a model substance, we used droplets generated at 1 Hz containing 1 mM Serotonin. Higher magnification objectives are highly beneficial since they typically provide wider numerical apertures; therefore, yielding higher photon counts. Also, they focus the excitation light at a smaller volume. It led us to the selection of 40× (NA=0.6, WD=3.00-4.20 mm) and 60× (NA=1.2, WD=280 µm) objectives. A comparison of the optical gain of these two objectives is presented in Figure 1A. According to the results in Figure 1A, we obtain a higher than ten-fold photon gain in the case of 60×.


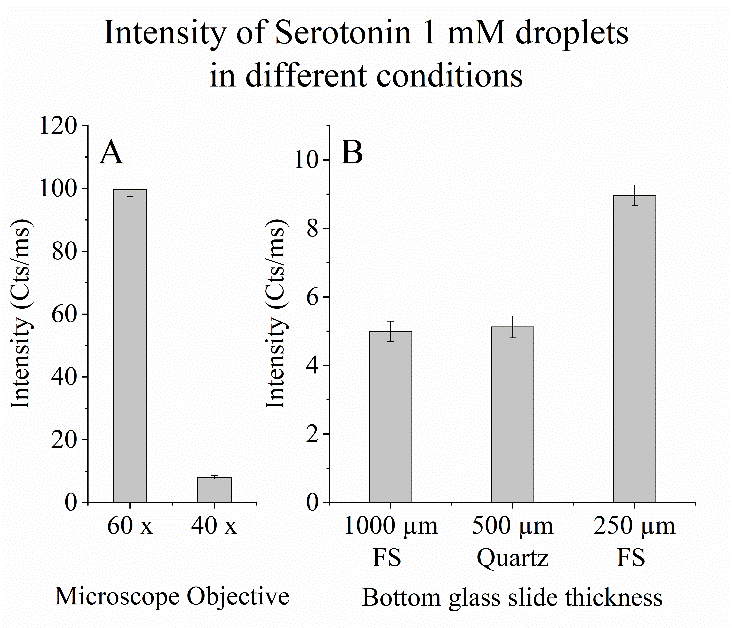


Figure S1 A: Comparison of photon count of two different objectives using the 250 µm FS + 20 µm PDMS bottom chip and 4.43 mW laser power on serotonin 1 mM droplets at 1 Hz. B: Effect of chip bottom thickness on photon counts using same conditions and the 40× objective. More than 100 droplets were counted for each measurement (n>100).

Notably, in an inverted microscopic arrangement, the excitation and emission light transmission is significantly affected by the bottom layer’s thickness and absorption coefficient. An ideal chip’s bottom layer is as thin as possible to ensure optimum optical transparency. In order to investigate the influence of the glass bottom-thickness on the optical gain, glass slides with 250 µm, 500 µm, and 1 mm with 20 µm of PDMS were investigated as bottom material. This set of experiments is carried out under similar conditions to the previous one. As an objective we choose the 40x lens since the working distance (3.0-4.2 mm) is compatible with thicker glass slides. The 60x lens cannot be used to investigate glass slides thicker than 250 µm since it has a limited low working distance of 280 µm. The corresponding results are given in Figure 1B.

As expected, among three different chips, the one with 250 µm bottom thickness shows a higher photon count (about 60 % higher) than the thicker chip bottoms. From these preliminary tests it follows that the highest sensitivities are achieved with chips with a 250 µm FS bottom coated with a 20 µm PDMS layer in combination with a 60× objective. In contrast, the combination of the 40× objective with the 1 mm FS+20 µm PDMS chip bottom is optically not the best selection but most physically stable. It is of most practical convenience to use 1 mm FS slides as they are not as fragile as the thinner slides and commercially widely available. Therefore, this combination of 1 mm FS+20 µm PDMS chip bottom was used for the sorting experiment chips. A summary of the interacting influences of lenses and chip variations is shown in Table 1.

Table 1. Comparison of chip variations. The optical gain is normalized by the maximum photon count.

| Chip type | | Objective compatibility | | Optical gain  (photon count) | Advantages | Disadvantages | Comment |
| --- | --- | --- | --- | --- | --- | --- | --- |
| FS or quartz thickness | PDMS thickness | 40× NA=0.6 WD=3.00 -4.20 mm | 60× NA=1.2 WD=280 µm |  |  |  |  |
| 1 mm | 20 µm | ✓ | Not  applicable | 55.7 % | Easy to handle;  physically stable | Poor optical gain | Most durable chip |
| 500 µm | 20 µm | ✓ | Not  applicable | 57.1% | Easy to handle;  physically stable | Poor optical gain | No unique physical or optical advantage |
| 250 µm | 20 µm | ✓ | ✓ | 100 % | Best optical transparency | Extremely fragile | Recommended only for low-photon count experiments |

# Laser power and droplet frequency optimization

To optimize the laser power, it is necessary to find a laser power that is high enough to achieve the highest possible number of fuorescence photons without damaging the chip.


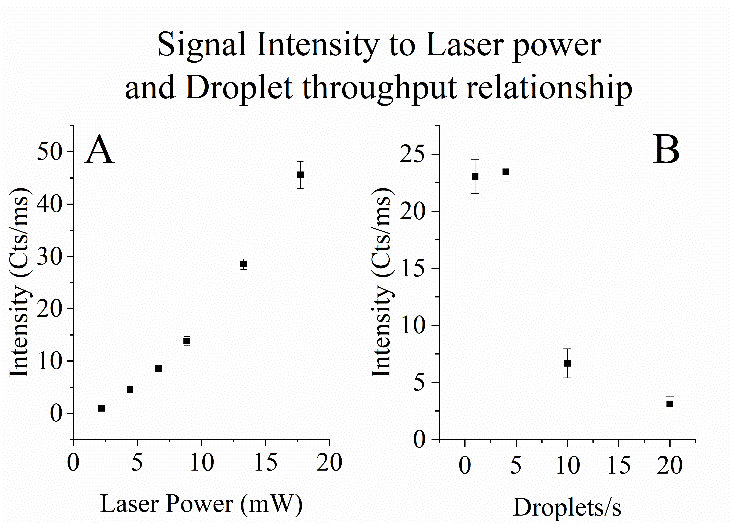


Figure S2.A: TPE photon count while increasing laser power on the 1 mm FS+20 µm PDMS chip with 1 Hz serotonin droplets of 1 mM concentration, using the 40× objective. B: Decline of photon count due to increased droplet frequency, applying similar conditions and 13.39 mW laser power. More than 100 droplets were counted for each measurement (n>100).

For the intended fluorescence lifetime measurements, it is important in this context to determine the minimum number of photons needed for an accurate determination of the fluorescence lifetimes by time-correlated single photon counting under TPE conditions at 532nm. To this end we performed a set of experiments for on-the-fly TCSPC measurements of droplets. Droplets of a 1 mM aqueous serotonin solution were generated with the chip (1 mm FS+20 µm PDMS) at a frequency of 1 Hz. The droplets were then analyzed with the 40x objective employing different laser powers. The results are presented in Figure 2A.

Figure 2A shows that the photon number rises almost linearly with increasing laser power. Interestingly, photobleaching was not observed up to 20 mW laser power, which is typically very high for fluorescence detection. This can be explained by the continuous flow of droplets and the small excitation spot of the two-photon excitation.

The results in Figure 2A show that a ~14 mW laser power yields a photon count of ~30 Cts/ms. This is more than sufficient to calculate average fluorescence lifetime with high accuracy from 1 mM serotonin droplets at 1 Hz while using the 1 mm FS+20 µm PDMS chip bottom and 40× objective. 4.43 mW was determined as sufficient laser power for sorting experiment as the standard deviation of the samples for 1 mM propranolol and serotonin droplets was determined to be <0.05 ns and <0.015 ns, respectively. However it needs to be mentioned, that the laser power to photon count relationship is strongly influenced by the precision of laser beam alignment and focusing.

To investigate the achievable throughput of the system, we varied the droplet frequency from 1 to 20 Hz in a series of experiments. For this, we again used 1 mM serotonin as droplet content and performed the detection at 13.39 mW laser power with otherwise similar parameters as for Figure 2A. As can be seen in Figure 2B, the photon number decreases significantly with increasing droplet frequency. This system is able to detect droplets with accurate fluores-cence lifetime at 10 Hz, but reaches its limits at 20 Hz, when the photon count is insufficient to accurately calculate the lifetimes.

# Fluorescence lifetime of dead and alive cells

Autofluorescence intensity and lifetime plotted simultaneously demonstrating fluorescence property of individual cells. In Figure 3, every dot represents a detected cell. Every cell has a photon-count, represented as the intensity on the vertical axis., and a calculated FLrepresented on the abscissa in the histogram. On the upper side, a histogram shows the average fluorescence lifetime distribution.





Figure S3 Representation of detected yeast cells regarding their photoncount and their avarge fluorescence lifetime.
